# Real-life experiences with galcanezumab and predictors for treatment response in Turkey

**DOI:** 10.1186/s12883-023-03467-1

**Published:** 2023-11-23

**Authors:** Pınar Yalinay Dikmen, Betül Baykan, Derya Uludüz, Aynur Özge, Elif Ilgaz Aydınlar, Burcu Polat, Necdet Karlı, Nermin Tepe, Neşe Çelebisoy, Hayal Ergin Toktaş, Buket Niflioğlu, Rahşan Karacı, Füsun Mayda Domaç, Ezgi Uludüz, Tuba Erdogan Soyukibar, Nevra Öksüz, Mustafa Ertaş

**Affiliations:** 1 School of Medicine, Neurology Department, Acibadem University, Büyükdere Caddesi. No: 40, Istanbul, 34390 Turkey; 2 Private practice, Istanbul, Turkey; 3 Cerrahpasa Medical Faculty, School of Medicine, Neurology Department, Istanbul University, Istanbul, Turkey; 4 Faculty of Medicine, School of Medicine, Neurology Department, Mersin University, Mersin, Turkey; 5 School of Medicine, School of Medicine, Neurology Department, Istanbul Medipol University, Istanbul, Turkey; 6 Faculty of Medicine, School of Medicine, Neurology Department, Uludag University, Bursa, Turkey; 7 Faculty of Medicine, School of Medicine, Neurology Department, Balikesir University, Balıkesir, Turkey; 8 Faculty of Medicine, School of Medicine, Neurology Department, Ege University, Izmir, Turkey; 9 Neurology Department, Bayindir Hospital, Istanbul, Turkey; 10 Neurology Department, University of Health Sciences, Istanbul, Turkey; 11 Koc University Medical School, Istanbul, Turkey; 12 Istanbul Faculty of Medicine, School of Medicine, Neurology Department, Istanbul University, Istanbul, Turkey

**Keywords:** Calcitonin gene-related peptide, CGRP, Galcanezumab, mAbs, Monoclonal antibody, Migraine, Migraine prevention, Anti-CGRP

## Abstract

**Background:**

The complexity of clinical practice extends far beyond the controlled settings of trials, and there is a need for real-world studies aimed at identifying which patients will respond to anti-CGRP monoclonal antibodies in different countries. This study aimed to investigate the efficacy and safety of galcanezumab in treating migraine in a real-life setting in Turkey, as well as identify predictors of treatment response.

**Methods:**

A total of 476 patients who diagnosed with migraine according to ICHD-3 criteria and treated with galcanezumab by headache specialists were voluntarily participated in this cross-sectional study. Galcanezumab is indicated for the prevention of migraine in adults who have at least 4 monthly migraine days in Turkey. All patients filled out a survey on Google Form that comprised 54 questions, addressing various aspects such as demographics, migraine characteristics, previous use of acute symptomatic medication, failures with preventive drug classes, comorbidities, most bothersome symptoms, as well as the interictal burden of migraine.

**Results:**

Among the participants, 89.3% reported that galcanezumab treatment was beneficial for them. A decrease in the frequency (80.0%), severity (85.7%), and acute medication usage for migraine attacks (71.4%) was reported with galcanezumab treatment. An adverse effect related to galcanezumab was reported in 16.3% of cases, but no serious adverse reactions were observed. Remarkably, 14.3% of participants reported no longer experiencing any headaches, and 18.9% did not require any acute treatment while receiving galcanezumab treatment. A logistic regression model showed that male gender, lack of ictal nausea, and previous failure of more than 2 prophylactic agents may predict the non-responders.

**Conclusions:**

The first large series from Turkey showed that galcanezumab treatment is safe and effective in most of the patients diagnosed with migraine by headache experts in the real-life setting. Patients reported a significant decrease in both ictal and interictal burden of migraine and expressed satisfaction with this treatment.

**Supplementary Information:**

The online version contains supplementary material available at 10.1186/s12883-023-03467-1.

## Background

Migraine is a chronic, prevalent, and disabling neurological disorder that can significantly impact individuals’ quality of life, causing social isolation and hindering daily activities [1]. Studies have addressed concerns raised by patients and clinicians regarding the limited efficacy of nonspecific prophylactic medications for individuals with migraine, as well as the potential adverse effects that can make them challenging to tolerate. These factors often contribute to poor adherence to treatment hindering the achievement of desired outcomes [2]. The development of monoclonal antibodies (mAbs) targeting the calcitonin gene-related peptide (CGRP) has opened a new era in the prevention of migraine.

Galcanezumab is a mAb that specifically targets the CGRP, which plays a crucial role in the underlying mechanisms of migraine. Four phase III, multinational, double-blind, randomized, placebo-controlled trials, named EVOLVE-1, EVOLVE-2, REGAIN, and CONQUER and subsequent studies have demonstrated the efficacy of galcanezumab in reducing mean monthly headache days (MHDs) compared to placebo over a duration of 3 to 12 months with an open-label extension. Furthermore, a consistent reduction in scores related to migraine-induced disability and the utilization of acute medications was observed across patients affected by both episodic migraine (EM) and chronic migraine (CM) following galcanezumab treatment.

However, the complexity of clinical practice extends beyond the controlled settings of pivotal trials and there is a growing number of real-world evidence (RWE) dedicated to identify the specific patient cohorts that will exhibit a positive response to anti-CGRP mAbs [3–7]. Several factors have been specified as predictors and persistence of mAbs’ response in different geographic regions. Various demographic and clinical factors including age, gender, body mass index (BMI), baseline migraine frequency and disability, pain severity and location, presence of allodynia, dopaminergic symptoms, response to triptans, psychiatric comorbidities, and personality traits have been reported as they may play a role in responsiveness to the treatment. However, the heterogeneity of these findings may be influenced by differences in the populations studied, sample sizes, study designs, and the specific clinical endpoints investigated [8–19].

The reasons for the geographic variability of mAbs’ treatment response are not entirely clear. However, one possible explanation is the differences in genetic and environmental factors that may influence the expression and regulation of CGRP. The findings pertaining to DNA methylation in genes responsible for encoding CGRP and its receptor at the peripheral level suggest a potential role of epigenetic alteration in migraine [20, 21]. For example, patients in North America may have different lifestyle factors or comorbidities that affect their response to treatment compared to patients in Europe or Asia. Additionally, differences in patient populations, including demographics and migraine characteristics, may contribute to differences in treatment response.

Despite the utilization of galcanezumab as a preventive treatment for migraine in Turkey since June 2021, there is currently a lack of published studies addressing the RWE of patients undergoing this treatment. The objective of this study was to assess the benefits of treatment in patients with migraine during both the ictal and the interictal periods, besides investigating potential predictive factors for galcanezumab treatment. To achieve this, we employed a survey created by using Google Forms.

## Methods

In this comprehensive cross-sectional study, participants were diagnosed by headache specialists located in diverse regions across Turkey, including those associated with universities, state hospitals, and private practices. Through networking at national conferences, we identified institutions and private practices where headache medicine specialists operate. These centers are known for their careful prescription and supervision of galcanezumab, as well as their regular follow-up and treatment of headache patients. Collaboration invitations for this study were extended by the first two authors and the senior author.

Galcanezumab is indicated for the prevention of migraine in adults who have at least 4 migraine days per month in Turkey. Treated patients fulfilled the requirements set by the regulatory agency of the Government of Turkey for galcanezumab treatment. The study included participants who were aged 18 years or older and had a diagnosed migraine according to International Classification of Headache Disorders, 3rd edition criteria with a minimum of one year of history [22]. The participants could have been diagnosed with medication overuse headache (MOH) and they might be either receiving preventive treatment or not. They started a preventive treatment with galcanezumab (240 mg initial dose followed by 120 mg monthly) between June 2021 and April 2023. Patient recruitment was initiated simultaneously at all centers in February 2023 and terminated simultaneously at the end of the 3-month study period. The patients who cannot understand and complete the survey questions due to cognitive difficulties or other specific communication problems were excluded from the study.

The study was conducted in accordance with the Declaration of Helsinki. Institutional review board approval was obtained from Acibadem University School of Medicine. All participants provided written consent after being informed about the study.

A total of 476 patients diagnosed with migraine volunteered to participate in this study. All patients completed a survey on a Google Form, which consisted of 54 questions (Supplement). The recruitment process involved two approaches led by headache specialists. Initially, physicians reviewed their medical records to identify patients who had previously received treatment with galcanezumab. Subsequently, these patients were contacted via phone and extended an invitation to participate in the study. In this first approach, a total of 166 patients who were treated with galcanezumab were enrolled by a physician who completed the Google form while conducting phone interviews with the patients. During the recruitment phase, an additional approach involved enrolling patients who regularly attended appointments with physicians. Using this approach, a total of 310 patients who had received galcanezumab treatment were included in the study. Out of the eligible participants, only 21 individuals declined to participate. Moreover, there were 61 registered patients who had received galcanezumab treatment whom we were unable to contact either by phone or email throughout the study period.

The Google form consisted of 12 sections (Table 1). Key information was obtained from physicians’ records for all participants, including the date of initial treatment, MHDs, and use of acute symptomatic medication in the previous month before galcanezumab. The records also provided details about migraine type (migraine with aura (MwA), migraine without aura (MWoA), migraine characteristics, accompanying symptoms, medical history, duration of the disease, classes of previous acute symptomatic medication, and previous failures with preventive drug classes.

**Table 1:** Sections and topics of survey

Section	Questions	Topics
Demographics	1–6, 13	Identity, age, gender, education, work status, physician
Comorbidities	8,9	Comorbid diseases and treatment
Clinical features of migraine	10,11	Type*, duration
Previous migraine treatment	12	Past preventive treatment/s,
Provider of galcanezumab	7	Reimbursement method
Information about Galcanezumab treatment	14,15,16,17, 20,21,22	Continuing and/or quitting of treatment, a number of injections, a last date of injection, a duration of treatment, a length of treatment,
Headache improvements with galcanezumab treatment	18,19, 30, 32, 36,37	A general thought about galcanezumab (beneficial or not), changes of headache frequency and severity, and frequency of acute medication use, MHDs, acute medication use in the past month
Side effects	23,24	Adverse effect/s
Previous clinical status of migraine	25,26,27, 28, 29	Pre-treatment MHDs, pain severity, acute medication use per month, classes of acute medication**
Patient- reported primary outcomes	31, 33, 35	Change rates (0-100 scale) of headache frequency, severity, acute medication use
Accompanying symptoms	38, 39, 40	Accompanying symptoms during migraine attacks and improvement rates in those symptoms, MBS
Interictal burden	41, 42, 43, 44, 45, 46, 47, 48, 49, 50, 51, 52, 53, 54	Mood, life quality, health, sleep, anxiety, control of migraine, sense of helpless, social or leisure activities, interpersonal relationships, attention, and concentration

MHDs: Monthly headache days, MBS: Most bothersome symptom, MwA: Migraine wiht aura, MwoA: migraine without aura, NSAIDs: Non-steroidal anti-inflammatory drugs

* Migraine wiht aura or migraine without aura or both

** Simple pain killers, NSAIDs, triptans, combination medications, ergotamine, metamizole, opioids, herbals, non-pharmacologic approaches

Regarding galcanezumab, participants were asked about the treatment duration, the method of reimbursement, number of injections, any adverse effects experienced, and the overall benefits derived from treatment. Throughout the recruitment phase, MHDs and the frequency of acute medication intake within the past month were also recorded to assess the participants’ current condition. The survey covered other topics such as the most bothersome symptom (MBS) and accompanying features of attacks as well as interictal burden of migraine.

The primary outcomes of the study focused on the percentage reduction from baseline in terms of MHDs (headache frequency), headache severity, and frequency of acute medication use as reported by patients receiving galcanezumab treatment (Questions 29,31,33). The secondary outcomes of the study were divided into five categories. First, patients evaluated the percentage of improvement regarding MBS and accompanying features with the treatment during their migraine attacks. Second, the rates of benefit from galcanezumab were divided into categories according to how patients responded to the treatment: 50–74% reduction, 75–99% reduction, and complete elimination of headaches (100% reduction). Third, when assessing the interictal burden of migraine, participants were asked whether their mood, quality of life, health, and sleep -specifically their sleep- improved with galcanezumab treatment. Furthermore, in a section focusing on the interictal burden of migraine, the patients assessed alterations in anxiety levels, migraine control, sense of helplessness, participation in social and recreational activities, interpersonal relationships, as well as attention and concentration. Fourth, we defined a positive response to galcanezumab as patients reporting at least a 50% reduction in headache frequency. An analysis was conducted to compare two groups: the nonresponsive group (< 50% response rate) and the responsive group (≥ 50% response rate), based on the reduction in headache frequency. The goal was to identify any demographic or clinical factors linked to the response to treatment. Fifth, a binary logistic regression analysis was performed to predict the likelihood of responding to galcanezumab.

### Statistical analysis

This is the primary analysis of these data. All the analyses were performed on the available data. No a priori statistical power calculation was conducted. The sample size was based on the available data. There were no missing data because the Google survey form was designed to prevent respondents from skipping required questions. Data were expressed as mean (SD) and median (minimum-maximum). We compared average scores using the unpaired t-test for continuous variables and chi-square test for categorical variables. All two-sided p-values were less than 0.05 which is considered statistically significant. We also used a binary logistic regression model to determine the baseline characteristics associated with a 50% response to galcanezumab. Statistical analyses were performed using Statistical Package for the Social Sciences (SPSS) Statistics for Windows, version 16 (IBM Corp.).

## Results

### Demographics of the cohort

The average age of the patients in our cohort was 42.9 (10.6) years, ranging from 18 to 77 years. The age distribution of our cohort was divided into four groups for further analysis: 18–34 years (22.5%), 35–49 years (49.2%), 50–64 years (25.6%), and > 64 years (2.8%). The average duration of migraine among the participants was 18.8 (10.8) years, ranging from 1 to 57 years. In terms of education, 70.1% (*n* = 335) of the participants had graduated from university, while 19.1% (*n* = 91) had completed high school. Regarding employment status, 65.5% of the patients were gainfully employed. In the study, 72.9% (*n* = 347) of the patients were purchasing galcanezumab themselves without any health insurance or social security, while 27.1% (*n* = 129) of the participants were receiving reimbursement for their treatments.

### Clinical features and comorbidities of migraine

In our cohort, the mean MHDs and number of days with acute medication were 14.7 (8.2) and 13.3 (8.0) per month before galcanezumab, respectively. Among the participants, 49.6% (*n* = 235) were classified as having CM, while 30.5% (*n* = 145) were classified as having high-frequency episodic migraine (HFEM). Among the CM group, 86.0% (*n* = 204) were also classified as having MOH according to ICHD-3 criteria [22].

Prior to galcanezumab treatment, 97.5%, of the patients (*n* = 464) reported experiencing moderate (27.1%) or severe (70.4%) headaches that had a significant impact on their quality of life.

**Fig. 1:**
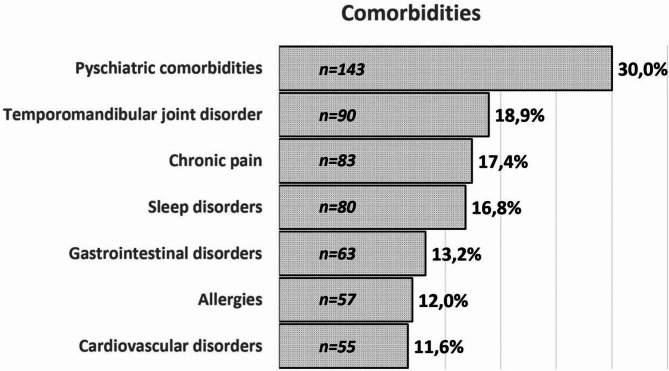
Comorbid conditions of the participants

In terms of migraine types, 12% (*n* = 57) of patients were experiencing pure MwA, 68% (*n* = 324) were defining MwoA attacks and both types of attacks were present in 33.8% (*n* = 161). Of all the participants, 33.8% (*n* = 161) had comorbid diseases, and 50% of them were receiving various medical treatments for their comorbid disorders (Fig. 1).

### Acute and prophylactic migraine treatment experiences

In our cohort, 97.7% (*n* = 465) of the participants had previously used various drugs for acute migraine attacks (Table 2). During enrollment for the study, 55.5% of the participants (*n* = 264) was currently receiving other prophylactic treatments for migraine. In our cohort, 15.8% of the participants (*n* = 75) did not have any prophylactic treatment experiences. However, 37.9% of patients had experiences with at least two or more preventive medication for migraine. Table 3 illustrates previous preventive treatment experiences which were reported by the participants.

**Table 2:** Acute treatment experiences of the participants

Medication classes	%, (*n*)
NSAIDs	55,5 (264)
Triptans	66.8% (318)
Paracetamol	23,5 (112)
Combination medications	23,3 (111)
Ergotamine	13.2% (63)
Metamizole	0.4% (2)
Tramadol	0.4% (2)

*n*: Number of subjects, NSAIDs: Non-steroidal anti-inflammatory drugs

**Table 3:** Medication classes which were previously used by the participants for their preventive treatments

Medication class	%, (*n*)
Antidepressant drugs	55.9, 266
Antiepileptic drugs	51.9, 247
Onabotulinum-toxinA	37.0, 176
Beta blockers	34.2, 163
Calcium channel blockers	29.2, 139
Nerve blocks	27.1, 129
Acupuncture	20.0, 95
Neural treatment	9.9, 47
Other mAbs imported from abroad	1.5, 7
Migraine surgery	1.5, 7

*n*: Number of participants, mAbs: Monoclonal antibodies

### Galcanezumab treatment experiences

During our study, galcanezumab treatment was continued by 54.2% (*n* = 221) of the patients. Among the participants, 89.3% (*n* = 425) reported that galcanezumab treatment was beneficial for them. The mean duration for the onset of the beneficial effect of galcanezumab was determined to be 1.0 (2.4) month. Indeed, 65.9% of patients (*n* = 280) reported experiencing benefits starting within the first month, followed by 17.2% (*n* = 73) in the second month, 9.4% in the third month, 2.8% (*n* = 12) in the fourth month, and the remaining 4.7% (*n* = 71) after the fifth month.

The length of treatment with galcanezumab prior to taking survey 7.97 ± 5.17 months, ranging from 1 to 24 months. Among the patients, 13.7% (*n* = 65) continued galcanezumab treatment for 6 months, 11.3% (*n* = 54) for 12 months, and 2.1% (*n* = 10) for 18 months. Among the 129 patients who discontinued galcanezumab, the median duration of treatment discontinuation was 6.6 (3.5) months, with a range of 1 to 18 months.

Patients were asked whether they had to discontinue galcanezumab treatment. In our cohort, 72.9% of the participants reported that they did not have to discontinue the treatment. However, 16.8% of the participants reported that they had to discontinue the treatment due to economic reasons, despite wanting to continue. Additionally, 8.6% of the participants reported discontinuing the treatment due to its ineffectiveness, while 1.7% cited side effects as the reason for discontinuation. Out of 129 participants who answered the question about having to restart galcanezumab treatment after discontinuation, 16.3% (*n* = 21) reported that they had to resume the treatment because their headaches worsened.

### Primary outcomes of the study

**Fig. 2:**
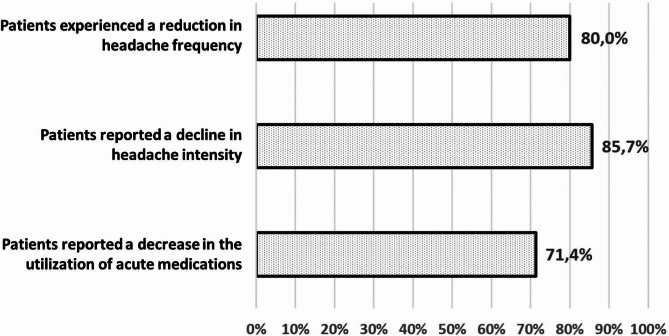
Primary outcomes of the study

The main outcomes assessed in this study included the percentage reduction in headache frequency, severity, and the need for acute treatment reported by patients after receiving galcanezumab treatment compared to their baseline. The results of our study indicated that 80.0% of the patients experienced a reduction in headache frequency, 85.7% reported a decline in headache intensity, and 71.4% reported a decrease in the utilization of acute medications (Fig. 2).

Within our study population, 14.3% (*n* = 68) of participants reported no longer experiencing any headaches (super-responders), and 18.9% (*n* = 90) of patients did not require any acute treatment while receiving galcanezumab treatment. The frequency of current headache and need for acute medication were evaluated by two questions. Mean MHDs and days using acute medication were reported as 4.8 (6.0) and 3.8 (5.4) by the patients, respectively.

### Secondary outcomes of the study

#### Most bothersome symptom and accompanying features

Before treatment, photophobia (83.8%, *n* = 399), phonophobia (82.8%, *n* = 394), nausea (75.8%, *n* = 361), headache aggravation by routine physical activity (67.0%, *n* = 319), osmophobia (55.0%, *n* = 262), and vomiting (32.1%, *n* = 153) were reported in various ratios by the patients during the attacks. Before the treatment, the MBS experienced during migraine attacks were reported as photophobia (40.3%, *n* = 185), followed by nausea (23.7%, *n* = 109), phonophobia (21.6%, *n* = 99), and vomiting (13.7%, *n* = 63). Table 4 presents data on the improvement of migraine attack-related symptoms following treatment with galcanezumab.

**Table 4:** The changes of accompanying symptoms in migraine attacks with galcanezumab treatment

Symptom in migraine attack	No previous complaint *n* (%)	No improvement *n* (%)	≤%50 improvement *n* (%)	>% 50–99 improvement *n* (%)	100% improvement *n* (%)
**Nausea**	86 (18.1)	56 (11.8)	37 (7.8)	155 (32.6)	142 (29.8)
**Vomiting**	280 (58.8)	22 (4.6)	14 (29.0)	46 (9.7)	114 (23.9)
**Photophobia**	51 (10.7)	84 (17.6)	69 (14.5)	166 (34.9)	106 (22.3)
**Phonophobia**	61 (12.8)	72 (15.1)	78 (16.4)	166 (34.9)	99 (20.8)
**Osmophobia**	177 (37.2)	65 (13.7)	43 (9.0)	112 (23.5)	79 (16.6)
Aggravation*	130 (27.3)	70 (14.7)	64 (13.4)	137 (28.8)	75 (15.8)

*N*: Number of participants, * Aggravation of by physical activity

#### The rates of benefit with galcanezumab

The response to galcanezumab treatment was defined as a reduction of at least 50% in reported MHD frequency by the patients. The patients who responded to treatment were categorized into three groups based on their response rates: 50–74%, 75–99%, and 100% (headache free). Table 5 demonstrates the demographic and clinical characteristics of these groups. One of the striking findings in the study, the unresponsiveness rate was higher in patients with CM associated w/o MOH compared to with MOH (25.8% vs.13.7%).

**Table 5:** The demographic and clinical characteristics of the participants

Variables	Non-responsive *n* (%)	50–74% of responsiveness *n* (%)	75–99% of responsiveness *n* (%)	Headache freedom *n* (%)
**All patients** (***n* = 476)**	64 (13.6)	129 (27.1)	215 (45.3)	68 (14.0)
**Females** (***n* = 384)**	46 (12.0)	103 (26.8)	179 (46.6)	56 (14.6)
**Males** (***n* = 92)**	18 (19.6)	26 (28.3)	36 (39.1)	12 (13.0)
**EM** (***n* = 241)**	28 (11.6)	62 (25.7)	107 (44.4)	44 (18.3)
**LFEM** (***n* = 79)**	10 (12.7)	23 (29.1)	33 (41.8)	13 (16.5)
**HFEM** (***n* = 145)**	17 (11.7)	39 (26.9)	65 (44.8)	24 (16.6)
**CM** (***n* = 235)**	36 (15.6)	67 (28.5)	108 (46.0)	24 (10.2)
**CM w/o MOH** (***n* = 31)**	8 (25.8)	8 (25.8)	13 (41.9)	2 (6.5)
**CM with MOH** (***n* = 204)**	28 (13.7)	59 (28.9)	95 (46.6)	22 (1.8)
**MwoA** (***n* = 324)**	42 (13.0)	90 (27.8)	146 (45.1)	46 (14.2)
**MwA** (***n* = 152)**	22 (14.5)	39 (25.7)	69 (45.4)	22 (14.5)
**Age of 18–34** (***n* = 106)**	15 (14.2)	39 (36.8)	38 (35.8)	14 (13.2)
**Age of 35–49** (***n* = 236)**	31 (13.1)	55 (23.3)	120 (50.8)	30 (12.7)
**Age of 50–64** (***n* = 121)**	15 (12.4)	32 (26.4)	53 (43.)8	21 (17.4)
**Age of > 65** (***n* = 13)**	3 (23.1)	3 (23.1)	4 (30.8)	3 (23.1)
**≤ 2 PPTF** (***n* = 297)**	28 (9.4)	73 (24.6)	142 (47.8)	54 (18.2)
**≥ 3 PPTF** (***n* = 179)**	36 (20.1)	56 (31.3)	73 (40.8)	14 (7.8)

EM: Episodic migraine, CM: Chronic migraine, MOH: Medication overuse headache, MWA: Migraine with aura, MwoA: Migraine without aura, PPTF: Previous prophylactic treatment failure

#### Interictal burden of migraine with galcanezumab

**Fig. 3:**
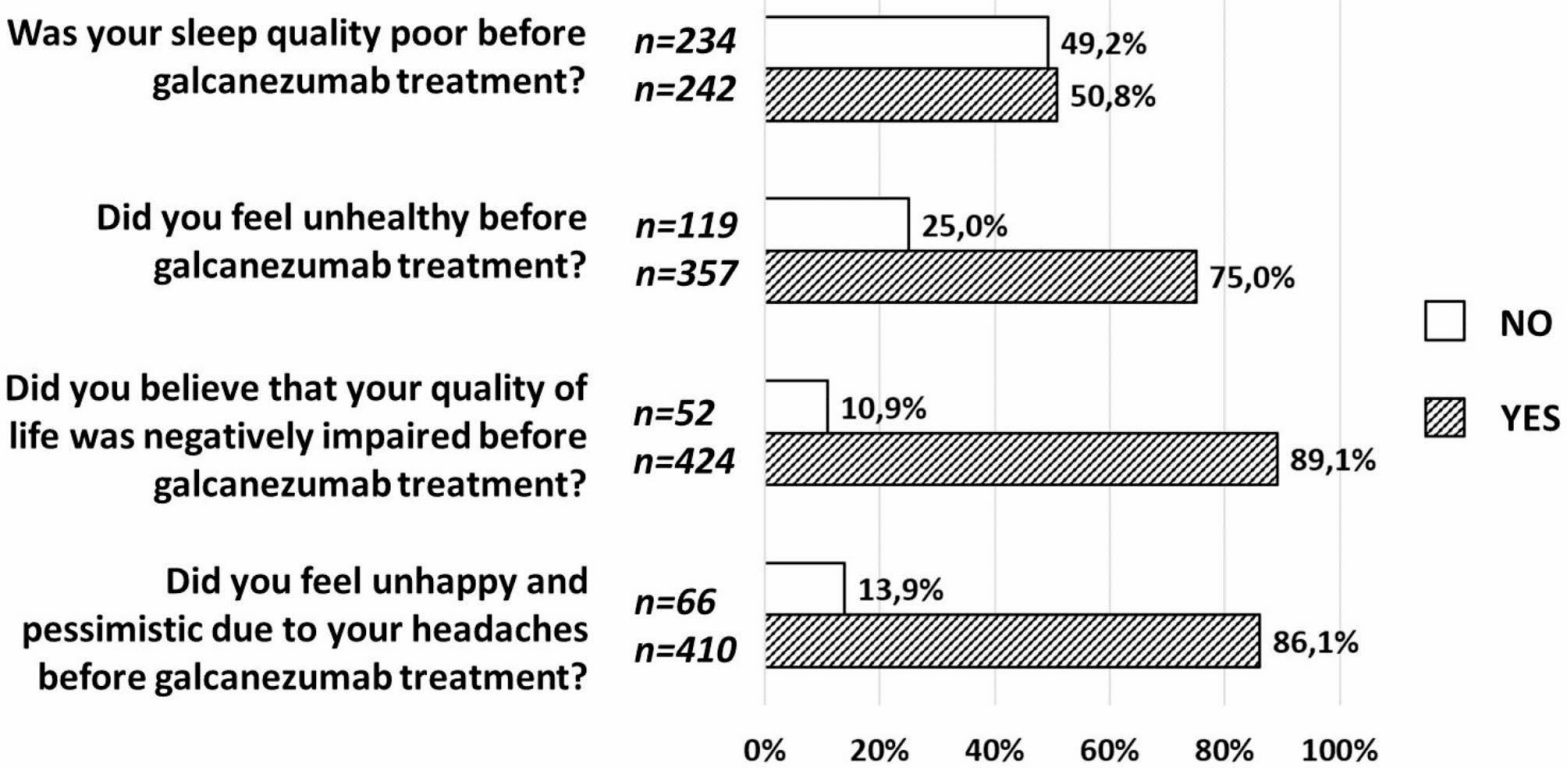
Changes interictal burden of migraine with galcanezumab treatment

Figure 3 shows the inter-ictal burden reported by patients, which reflects the impact of migraine on various aspects of their lives between attacks. In our cohort, 89.1% of patients reported a poor quality of life and 86.1% of those felt unhappy because of migraine before galcanezumab treatment. Moreover, 75.0% of participants identified themselves as unhealthy due to migraine.

**Fig. 4:**
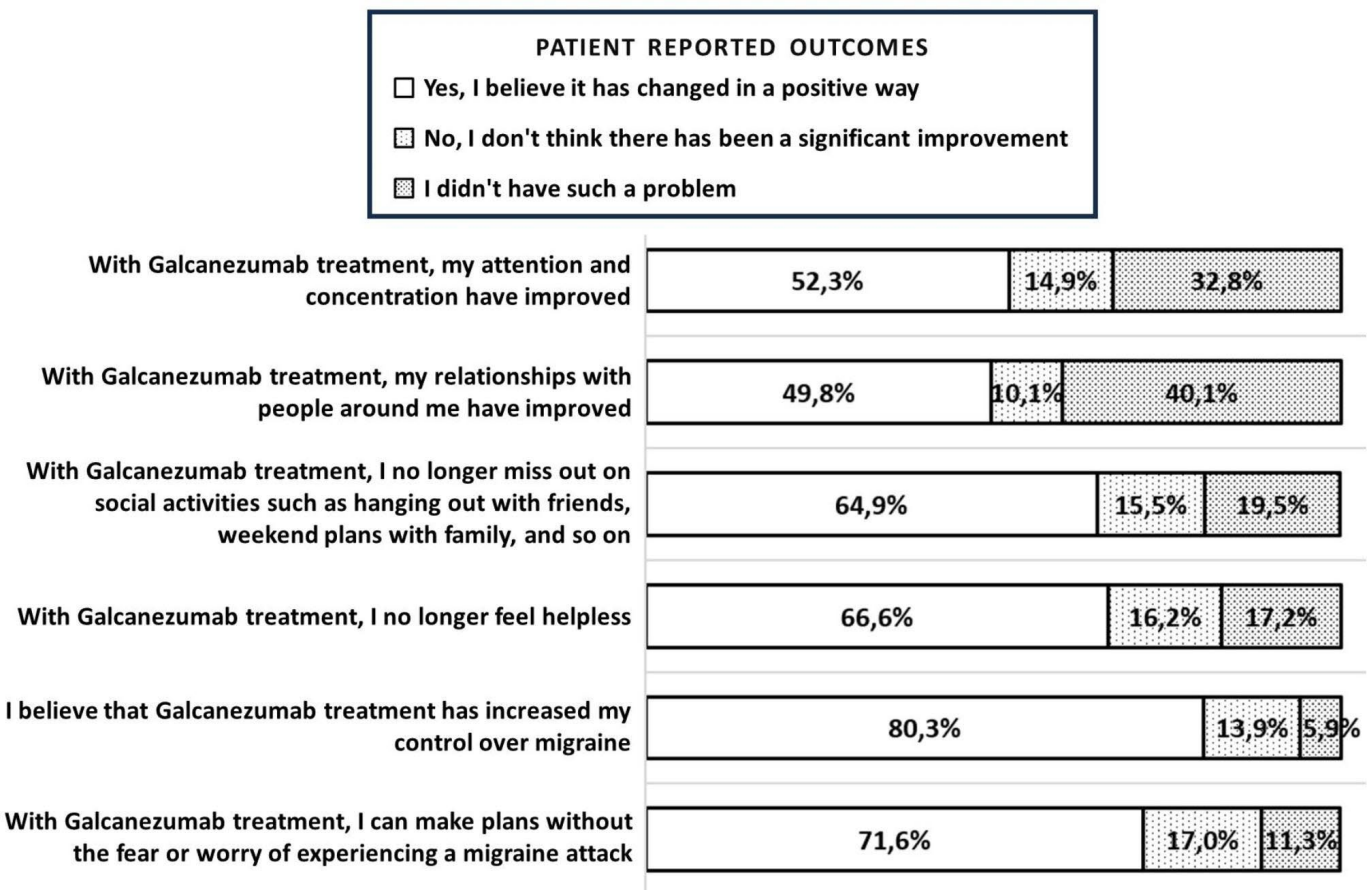
Patient reported outcomes regarding galcanezumab treatment

Figure 4 illustrates the responses reported by the participants, indicating the extent to which galcanezumab influenced different aspects of migraine interictal burden.

#### Comparison of nonresponsive (<%50 response rate) vs. responsive (≥ 50% response rate) groups based on a decrease of headache frequency

To determine the factors influencing the response to galcanezumab treatment, the demographic and clinical characteristics of these two groups were statistically compared. Demographic factors did not show a significant difference between the responsive and nonresponsive patients: age groups (*p* = 0.749), gender (*p* = 0.062). Among the clinical characteristics, only the variable of “previous ≥ 3 prophylactic treatment failures” (*p* = 0.001) was identified as a variable influencing headache frequency between the two groups. However, other clinical factors, such as aura (*p* = 0.667), duration of migraine (*p* = 0.720), CM (*p* = 0.282), MOH (*p* = 0.893), did not exhibit a statistically significant difference between the two groups. Comorbid conditions did not differ between nonresponsive patients and responsive ones; chronic pain without migraine (*p* = 0.066), comorbid psychiatric disorders (*p* = 0.660), and comorbid sleep disorders (*p* = 0.72). Moreover, accompanied features like ictal nausea (*p* = 0.058), ictal photophobia (*p* = 0.855), ictal phonophobia (*p* = 0.479), ictal osmophobia (*p* = 0.105) as well as headache aggravation by routine physical activity (*p* = 1.000) did not show statistically significance between two groups.

#### Logistic regression to predict for the response after galcanezumab treatment

A binary logistic regression model was employed to predict the response to galcanezumab by entering significant and nearly significant promising variables selected from the preceding analyses. The model revealed that gender (OR:0.636; CI:0.331–1.219), ictal nausea (OR:0.598; CI:0.324–1.104) and previous failure of more than two prophylaxes (OR:2.521; CI:1.470–4.326) can distinguish non-responders (Table 6). With a cut off value of 0.5 and a -2 log likelihood ratio of 358.7 (Cox Snell R-square: 0.035), the model successfully classified 86.6% of the patients correctly as responders or non-responders.

**Table 6:** Logistic regression model results for differentiating the ≥ 50% response rate

Variables	B	SE	Wald	Df	Sig	OR	95% C.I. Lower-upper	
**Gender**	-0.453	0.332	1.860	1	0.173	0.636	0.331	1.219
**≥ 3 previous preventives**	0.925	0.275	11.273	1	0.01	2.521	1.470	4.326
**Ictal nausea**	-0.514	0.313	2.703	1	0.100	0.598	0.324	1.104
**Constant**	1.604	0.212	57.085	1	0.000	4.971		

Df: Degrees of freedom, Sig: Significance, OR: Odds Ratio, C.I.: Confidence Interval

#### Side effects

**Fig. 5:**
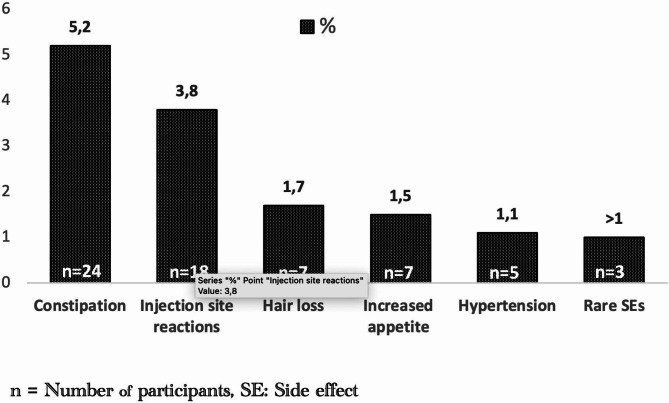
Adverse reactions related to galcanezumab reported by the participants

Overall, an adverse effect related to the treatment was reported in 16.3% (*n* = 76) of cases. Figure 5 shows side effects reported by the participants regarding galcanezumab treatment. However, no serious adverse reactions were observed. Rarely reported side effects (<%1) were palpitations, nausea, vertigo, abdominal pain, joint pain, fatigue, sleep disturbances, and decreased libido. Most reported side effects were managed by physicians with advice. In our cohort, only 8 patients (1.7%) discontinued treatment due to side effects.

## Discussion

In our large-sized RWE study, mean MHDs for the participants was 14.7 (8.2) before starting galcanezumab treatment, and 55.5% of them were under prophylactic treatment during the enrollment process. Three-quarters of the patients felt unhappy and unhealthy before treatment and believed that their quality of life was negatively impaired due to migraine. During the survey, 54.2% of the patients were currently using galcanezumab, and mean MHDs reduced to 4.8 (6.0) with treatment. Our findings showed that 80.0% of the patients reported a decline in the frequency of their headaches, 85.7% reported a reduction in intensity of their headaches, and 71.4% reported a decrease in the frequency of acute medication use with galcanezumab. Notably, 14.3% of the participants stated that they had no headaches (super responders), and 18.9% reported that they had never used any medication for acute attacks while receiving galcanezumab treatment. Photophobia (40.3%) was the most common reported MBS before galcanezumab. However, the most significant improvement was observed in nausea (62.6%) after treatment. In our study, the two groups with the highest non-responder rates were those with CM without MOH (25.8%) and those who had tried and failed with at least more than 2 prophylactic treatments (20.1%). The predominant factor influencing treatment response was the history of prior unsuccessful attempts. Logistic regression analysis to predict unresponsiveness to the treatment showed that male gender, lack of ictal nausea, and previous failure of ≥ 3 prophylactic drugs can differentiate the non-responders. Moreover, regarding the burden of migraine, 80.0% of our patients reported an improvement in their control over migraine with galcanezumab.

### Real-world evidence studies

A systematic analysis on RWE studies of mAbs against the CGRP-pathway reveals significant heterogeneity and/or a lack of predefined primary outcomes, objective definitions, and longitudinal monitoring in RWE studies on preventive treatments for migraine [23]. On the other hand, RWE studies play a crucial role in assessing the effectiveness of treatments, especially for patients with comorbidities who may not have been included in randomized clinical trials (RCTs). The observed heterogeneity in these studies can reflect variations in clinical practice across different countries and populations. In our cohort, 72.9% were self-funded galcanezumab. Allocating financial resources to this medication, it might be considered as evidence of the treatment’s effectiveness. Additionally, RWE studies, including our own data, have demonstrated the safety and effectiveness of these treatments in elderly migraine patients aged over 65 years and those with comorbid conditions. In our cohort with an average age of 42.9 years-old, just a small number of our patients (*n* = 13) were over 65 years old, which is consistent with real-life clinical experience. Although the size of our elderly sample was restricted, preventing us from making definitive assertions regarding treatment safety, it is noteworthy that we observed no complications concerning side effects aligning with findings from prior studies [24].

### RWE in Medication overuse headache and chronic migraine

Targeting the CGRP pathway with mAbs presents a significant benefit in efficiently addressing in patients with MOH, eliminating the necessity for potentially difficult withdrawal attempts that patients often find challenging [25, 26]. Certainly, our findings affirm the efficacy of galcanezumab in the management of both CM and MOH, consistent with outcomes reported in earlier, smaller RWE studies. Within our study cohort, 86% of CM patients exhibited MOH, with a non-responsive treatment rate of 13.7%, whereas this rate increased to 25.8% among those without MOH. Furthermore, our results suggest that a decrease in the necessity for acute treatment was observed in 69% of our patients, while 18.9% refrained from using any medication for managing acute attacks throughout their galcanezumab treatment period. Hence, drawing from our data, it can be inferred that galcanezumab therapy effectively diminishes the frequency of acute attacks and reduces the necessity for acute attack medications, while also exhibiting a favorable influence on MOH. Our patients’ real-world experiences align well with the outcomes of RCTs on galcanezumab’s effectiveness in preventing both EM and CM [27].

### Prediction of the response for mAbs

Several factors have been identified as predictors and persistence of anti-CGRP mAbs response in different geographic regions. In Korea, a medium sized study (*n* = 104) showed that chronic daily headache, presence of depression, and absence of accompanying symptoms of migraine were significant predictors of a poor response for patients with CM to galcanezumab, besides previously unsuccessful preventive medication classes [16]. A study from Europe reported that greater MMDs, a higher baseline Numeric Rating Scale (NRS) score, and a higher number of unsuccessful attempts at preventive treatments were associated with an unfavorable prediction for CM relief within the first year of galcanezumab treatment [25]. The only factor that exhibited a statistical difference between the responsive and nonresponsive groups in our study was the previous failure of at least two prophylactic treatments. In addition to that, individuals who will positively respond to preventive treatment for both CM and EM using galcanezumab could be predicted through the presence of pre-treatment non-ictal cephalic allodynia [9].

According to a prospective cohort study in Italy, triptan response, lower BMI, and MMD ≥ 50% reduction rate in the first month were identified as predictive factors for a persistent response [19]. Another Italian multicenter RWE study, using an observational, longitudinal cohort design, sought to assess the rapid response to galcanezumab and revealed that a favorable response to galcanezumab within the initial 3 months of therapy in patients with CM might be linked to factors such as unilateral pain, effective response to triptans, and a lower BMI [28].

Another prospective RWE study indicated that experiencing daily headaches, having depression, and lacking accompanying symptoms of migraine (such as nausea, vomiting, and sensitivity to light) emerged as noteworthy indicators of an unsatisfactory response, interestingly [16]. Zecca et al. showed that factors associated with a 75% responder rate for Erenumab included age at migraine onset, the count of unsuccessful preventive medications, and the MIDAS score [29].

The clinical assessment of super-responders (SR) and non-responders (NR) to anti-CGRP monoclonal antibodies is also of interest and a previous study focusing on this issue revealed that SR individuals, compared to NR, more frequently reported symptoms like vomiting (SR 48% vs. NR 18%; *p* = 0.031) and characteristic migraine features such as unilateral location, throbbing nature, sensitivity to light, and nausea. A subjectively favorable response to triptans was notably higher in SR (90%) compared to NR (60%, *p* = 0.010). NR patients experienced a higher occurrence of CM (NR 92% vs. SR 52%; *p* = 0.001), MOH (NR 58% versus SR 28%; *p* = 0.024), and concurrent depression (NR 65% vs. SR 28%; *p* = 0.005) [17].

It should be noted that some of these studies included multiple molecules, different inclusion criteria, and generally had smaller patient numbers, which can lead to heterogeneity in the results and contribute to variations in the identified predictors. Our study is one of the largest groups among RWE studies, specifically focusing on galcanezumab. We found that the male sex and absence of nausea may indicate a poor response to galcanezumab treatment. Nausea is a frequent and incapacitating symptom experienced by individuals with migraine. [30]. While the exact cause of nausea in migraine is not fully understood, we hypothesize that variations in the functional connections between trigeminal neurons and the nucleus tractus solitarius may contribute to its significance along with the possible changes of chronicification of migraine.

The pathophysiological mechanisms of migraine in males are not yet fully understood and appear to be complex and multifactorial. Neurochemical imbalances, specifically involving serotonin dysregulation, genetic and hormonal influences may play a role in shaping the clinical characteristics and severity of migraines in males. Additionally, central sensitization and cortical spreading depression, which are key processes in migraine pathophysiology, may manifest differently in males compared to females, resulting in distinct clinical presentations and symptoms [31]. Further research is needed to unravel the specific mechanisms underlying migraine in males and to develop targeted treatment approaches for this population.

### Side effects of galcanezumab in RWE

In our study, adverse events were infrequent, with constipation (5.2%) being the most common reported, followed by injection site reactions (3.8%). These findings align with previous RWE studies. Importantly, our large-scale study did not identify any significant safety concerns, and the adverse events profile remained consistent with the findings of published research in our country [32].

### Adherence to treatment and benefit

In our study, 16.8% of patients discontinued treatment due to financial reasons, 8.6% due to ineffectiveness, 1.7% due to side effects, and 72.9% reported continuing their treatment. These results indicate that galcanezumab exhibited good tolerability and a favorable long-term treatment adherence rate among Turkish patients.

The mean duration of onset of the beneficial effects of galcanezumab from the initiation was 1.0 (2.4) months, with 65.9% of patients (*n* = 280) reported experiencing benefits at the first month. Galcanezumab’s rapid onset of effect has been previously reported, demonstrating a significant impact as early as the first week after administration [33]. Our findings further support this data, indicating a lower percentage of slower responders.

A previous study reported that 83% (*n* = 38) of patients expressed dissatisfaction after a mandatory treatment break from a CGRP mAbs. This dissatisfaction was evidenced by an increase in monthly migraine days and the need for acute medication intake during the treatment break [34]. In our study, 129 patients who answered the question about restarting treatment after discontinuing galcanezumab, 16.3% (*n* = 21) reported that they had to restart the treatment. These findings suggest that interrupting therapy had a negative short-term impact on migraine patients, leading to worsened migraine frequency and increased reliance on acute medications. Furthermore, it has been observed that re-initiation of treatment with CGRP(-R) mAbs after a drug holiday results in a significant reduction in migraine frequency, decreased medication usage, and improvement in quality of life [35]. This highlights the positive effects of re-initiating CGRP(-R) mAbs after a treatment break, leading to better migraine management and overall well-being for patients.

### Improvement of burden of migraine

By prioritizing the patients’ viewpoints, we gained valuable insights into their personal assessment of the treatment’s success. Before galcanezumab treatment, over 75% of the patients described themselves as unhappy, unhealthy, and having impaired quality of life due to migraine. Additionally, more than 50% of the patients experienced sleep disturbances. However, after treatment, 80.0% of the patients reported an increase in their control over migraine, and 71.6% mentioned being able to plan activities without fear. Our data supports that galcanezumab reduces the interictal burden of migraine and improves the quality of life. Moreover, a study conducted in Spain suggests that migraine patients receiving galcanezumab express significantly higher levels of satisfaction compared to other preventive therapies, with meaningful reductions in migraine frequency, impact, and disability [36]. These findings further indicate the positive impact of galcanezumab on improving patients’ overall well-being and quality of life.

### Strengths and weaknesses

Our study, like other RWE studies, possesses both strengths and weaknesses. Notably, one of the strengths of our study is that it is the first multicenter study with a high number of participants from Turkey. This is particularly significant as Turkey has higher prevalence and incidence rates of migraine compared to other regions, making our study’s data valuable in providing insights into the management and treatment of migraine in this specific population [37]. Most of our patients have a high socioeconomic and education status and they are paying for galcanezumab treatment themselves. This may have positively influenced their expectations regarding the treatment, but they are individuals who express themselves accurately and have close collaboration with their headache experts. In our study, the benefit of treatment was analyzed in different subgroups at the rates of 50%, 75%, and 100% benefit, carefully.

One of the weaknesses of our study is a cross-sectional design that evaluates the treatment success of galcanezumab based on the benefit rates reported by patients to their physicians, but lack of a prospective headache diary that tracks monthly headache or monthly migraine days. Due to the multicenter nature of our study, some physicians may have performed detoxification for patients with MOH before or after the treatment. Similarly, physicians may have discontinued the previous prophylactic medication for patients starting galcanezumab or after achieving migraine control. These differences in practice, while reflecting the RWE of patients, may have played a role in the evaluation of the effectiveness of galcanezumab treatment. In our cohort, we have patients who have been receiving the treatment for 28 months as well as those who have received it for a short duration. Considering that migraine is a fluctuating disease, the cross-sectional observations of patients in such a heterogeneous group may not fully reflect the overall benefit of the treatment. Limitations of this cross-sectional RWE study also include potential selection bias and the lack of a control group. Lastly, the findings related to burden of migraine were based on patient self-reports, and standardized clinical scales or patient-reported outcome measures were not utilized in the study.

## Conclusions

Our findings revealed that previous treatment failures played a pivotal role in determining treatment responsiveness to galcanezumab. Additionally, our study suggests potential gender-specific mechanisms underlying the pathophysiology of migraine related to CGRP treatment response, which warrants further investigation. The improvement in migraine control for 80.0% of patients highlights the potential of galcanezumab in enhancing the quality of life for migraine sufferers. These results also provide valuable insights for identifying patients who may benefit the most from this treatment. However, it is important to acknowledge the limitations of our study, including its reliance on patient-reported cross-sectional data. Future research with larger and more diverse populations could further validate these results and contribute to a deeper understanding of galcanezumab’s efficacy and impact on patients with migraine.

### Electronic supplementary material

Below is the link to the electronic supplementary material.

Supplementary Material 1: Appendix: Google form

## Data Availability

The datasets of the current study are available from the corresponding authors upon reasonable request.

## Abbreviations

mAbs: Monoclonal antibodies
CGRP: Calcitonin gene-related peptide
MHDs: Monthly headache days
EM: Episodic migraine
CM: Chronic migraine
RWE: Real-world evidence
BMI: Body mass index
MOH: Medication overuse headache
MwA: Migraine with aura
MWoA: Migraine without aura
MBS: Most bothersome symptom
SPSS: Statistical Package for the Social Sciences
RCTs: Randomized clinical trials
SR: Super-responders
NR: Non-responders
CGRP(-R): Re-initiation of treatment with CGRP
MIDAS: Migraine disability assessment score
