# Supplementary material for: Real-life experiences with galcanezumab and predictors for treatment response in Turkey
Source: BMC Neurol. 2023 Nov 23;23:418. doi: 10.1186/s12883-023-03467-1 (PMC10666377; doi:10.1186/s12883-023-03467-1)
Supplement: Supplementary file 1 — Supplementary Material 1: Appendix: Google form [file 12883_2023_3467_MOESM1_ESM.docx]

EMGALITY (GALCANEZUMAB) EXPERIENCE SURVEY

In this survey, we would like to know your experiences with Emgality (Galcanezumab) that you have used. These findings will provide valuable insights for future applications. Thank you for your participation.

1. Would you write a first letter of your name?
2. Would you write a first letter of your surname?
3. What is your gender?

Male

Female

1. Would you write a date of birth?
2. What is your education level?

Primary school

High school

University

1. Do you work?

Yes

No

1. How do you supply Emgality (Galcanezumab)?

I am paying my medication myself.

I am getting my medication through health insurance.

1. Do you take any other medication regularly besides painkillers for migraine while using Emgality (Galcanezumab)? Please write down the name of medication(s) you are taking.

I do not take any medicine regularly.

……

1. Do you have any other medical condition besides migraine? If yes, please select all that apply.

No. I don’t have any other medical condition besides migraine.

Yes, I have below condition(s).

Chronic pain (fibromyalgia, joint pain, lumbago, another pain)

Psychiatric disease (depression, anxiety disorder)

Allergy (asthma, psoriasis, allergic flu, drug allergy)

Sleep disorder (restless leg syndrome, insomnia, sleep apnea)

Gastrointestinal diseases (reflux, ulcer, irritable bowel syndrome)

Heart and vessel diseases (diabetes mellitus, insulin resistance, hypertension, high cholesterol level)

Vertigo

Bruxism

Others

1. Would you describe your type of migraine?

Migraine with aura

Migraine without aura

I have both types of attacks.

1. How many years do you have migraine?
2. Have you used any preventive treatments for migraines in the past, apart from Emgality (Galcanezumab)? Please write down the name of medication(s) you were taking. Please select all that apply.

I did not use any preventive treatments before Emgality (Galcanezumab).

I was taken ….

I was treated by Botox,

I was treated by nerve block(s), acupuncture,

I was treated by neural therapy.

Others

1. Please write the name of your physician who started to Emgality (Galcanezumab) treatment.
2. Are you continuing with Emgality (Galcanezumab) treatment?

Yes

No

1. How many times have you had Emgality (Galcanezumab) injection in total? (Two injections that the first application is equal one injection)
2. What was the date of last Emgality (Galcanezumab) injection?
3. How many months did you use or are you using ‘Emgality’?
4. Do you think that the Emgality (Galcanezumab) treatment is beneficial for you?

Yes

No

1. In which month did you start to benefit from Emgality (Galcanezumab)?
2. Did you have to quit to Emgality (Galcanezumab)?

I did not have to quit the treatment.

I quitted the treatment because it was ineffective.

I had to quit the treatment due to adverse effects.

I wanted to continue my treatment, but I could not access the medication because of economic reasons.

1. How many months did you use Emgality (Galcanezumab) treatment before stopping it?
2. If your Emgality (Galcanezumab) treatment was successful, and you stopped the treatment with your physician’s suggestion, did your headaches worsen, and did you have to start again?

Yes

No

It is not related to me.

1. Did you have any adverse effect/s due to Emgality (Galcanezumab)?

Yes

No

1. Please write down any adverse effect/s due to Emgality (Galcanezumab).
2. How many days per month (30 days) did you have headache before starting Emgality (Galcanezumab) treatment (prior to the injection)?
3. Which one would you choose about the pain severity of your headache attacks before starting Emgality (Galcanezumab) treatment (prior to the injection)
   It's mild, not bothersome, and doesn't affect my daily activities.

It’s moderate, bothersome, and doesn't affect my daily activities.

It’s severe, I can’t perform my daily.

1. Before starting Emgality (Galcanezumab) treatment (prior to the injection), on average, how many days per month (30 days) were you using medication for pain relief?
2. Which group of painkillers did you use? (You can select more than one options.)

I did not use any painkiller.

Acetaminophen (Paracetamol) (Parol, Minoset etc.)

Combine painkillers (Including caffeine, codeine)

Non-steroidal anti-inflammatory drugs (Apranax, Arveles, Majezik,Brufen, Cataflam, Voltaren etc.)

Ergotamine (Avamigran, Cafergot)

Triptan (Relpax, Imigran, Migrex, Migreout)

Other (Please secify)

1. Please write the names of the painkillers you use when the headache comes). (If you use any)
2. Did your headache frequency change with Emgality (Galcanezumab) treatment?

I have never had a headache with Emgality (Galcanezumab) treatment.

My headache frequency has not changed with Emgality (Galcanezumab) treatment.

My headache frequency decreased with Emgality (Galcanezumab) treatment.

My headache frequency increased with Emgality (Galcanezumab) treatment.

1. When you consider the Emgality (Galcanezumab) treatment, how much did your overall headache frequency decrease in percentage? (Please provide a value on a scale of 0 to 100. For example, if it was 100 became 10, it means it decreased by 90%)
2. Did your headache severity change with Emgality (Galcanezumab) treatment?

My headache severity has not changed with Emgality (Galcanezumab) treatment.

My headache severity decreased with Emgality (Galcanezumab) treatment.

My headache severity increased with Emgality (Galcanezumab) treatment.

1. When you consider the Emgality (Galcanezumab) treatment, by what percentage do you think your headache severity has decreased in general? (Please provide a value on a scale of 0 to 100. For example, if it became 10 from 100, it means it decreased by 90%)
2. Did your frequency of pain killer usage for controlling headache change with Emgality (Galcanezumab) treatment?

My frequency of pain killer usage for has not changed with Emgality (Galcanezumab) treatment.

My frequency of pain killer usage for decreased with Emgality (Galcanezumab) treatment.

My frequency of pain killer usage for increased with Emgality (Galcanezumab) treatment.

I have never used any painkillers with Emgality (Galcanezumab) treatment.

1. When you consider the Emgality (Galcanezumab) treatment, by what percentage do you think your frequency of pain killers has decreased in general? (Please provide a value on a scale of 0 to 100. For example, if it became 10 from 100, it means it decreased by 90%)
2. During the Emgality (Galcanezumab) treatment, how many days in the last 30 days did you have pain?
3. During the Emgality (Galcanezumab) treatment, how many days in the last 30 days did you used pain killer for your headache?
4. Which of the following symptom/s is accompanying your migraine attacks? (You can select multiple options)

Nausea

Vomiting

Photophobia

Phonophobia

Osmophobia

Increasing headache severity with physical activity

1. Which symptom bothers you the most during a migraine attack? Please select only one option.
   Nausea

Vomiting

Photophobia

Phonophobia

1. In what direction did the following symptoms change during your migraine attacks after Emgality (Galcanezumab) treatment?

|  | Already absent | Same degree | Decreased by ≤ %50 | Decreased by %50 | Totally improved |
| --- | --- | --- | --- | --- | --- |
| Nausea |  |  |  |  |  |
| Vomiting |  |  |  |  |  |
| Photophobia |  |  |  |  |  |
| Phonophobia |  |  |  |  |  |
| Osmophobia |  |  |  |  |  |
| Increasing headache severity with physical activity |  |  |  |  |  |

1. Did you feel unhappy and pessimistic due to your headaches before Emgality (Galcanezumab) treatment?

Yes

No

1. What percentage did you improve in your unhappiness and pessimism with Emgality (Galcanezumab) treatment? (Enter 0 if no improvement) What percentage do you think your unhappiness and pessimism with Emgality (Galcanezumab) treatment? (Please provide a value on a scale of 0 to 100. For example, if it became 10 from 100, it means it imroved by 90%)
2. Do you believe that your quality of life was negatively impaired before Emgality (Galcanezumab) treatment?

Yes

No

1. What percentage did you improve in your quality of life with Emgality (Galcanezumab) treatment? (Please provide a value on a scale of 0 to 100. For example, if it became 10 from 100, it means it improved by 90%)
2. Did you feel unhealthy before Emgality (Galcanezumab) treatment?

Yes

No

1. What percentage of did you improve in your health with Emgality (Galcanezumab) treatment? (Please provide a value on a scale of 0 to 100. For example, if it became 10 from 100, it means it improved by 90%)
2. Do you believe that was your sleep quality poor before Emgality (Galcanezumab) treatment?

Yes

No

1. What percentage of did you improve in sleep quality with Emgality (Galcanezumab) treatment? (Please provide a value on a scale of 0 to 100. For example, if it became 10 from 100, it means it improved by 90%)

**We are curious about your experience with Emgality** (Galcanezumab) **treatment. Please answer the following questions based on your feelings and experiences related to the treatment.**

1. I can make plans without the fear or worry of experiencing a migraine attack thanks to Emgality (Galcanezumab) treatment.

I didn't have such a problem already.

Yes

No

1. I believe that Emgality (Galcanezumab) treatment has increased my control over migraine.

I didn't have such a problem already.

Yes

No

1. With Emgality (Galcanezumab) treatment, I no longer feel helpless.

I didn't have such a problem already.

Yes, I don’t feel it.

No, I still feel helpless.

1. Thanks to Emgality (Galcanezumab) treatment, I no longer miss out on social activities such as hanging out with friends, weekend plans with family, and so on.

I didn't have such a problem already.

Yes

No

1. My relationships with people around me have improved with Emgality (Galcanezumab) treatment.

I didn't have such a problem already.

Yes, it has improved.

No, it didn't get better.

1. With Emgality (Galcanezumab) treatment, my attention and concentration have improved.

I didn't have such a problem already.

Yes, it has improved.

No, it didn't get better.
